# Nanotube-structured Na_2_V_3_O_7_ as a Cathode Material for Sodium-Ion Batteries with High-rate and Stable Cycle Performances

**DOI:** 10.1038/s41598-018-35608-9

**Published:** 2018-11-21

**Authors:** Naoto Tanibata, Yuki Kondo, Shohei Yamada, Masaki Maeda, Hayami Takeda, Masanobu Nakayama, Toru Asaka, Ayuko Kitajou, Shigeto Okada

**Affiliations:** 1 Department of Advanced Ceramics, Nagoya Institute of Technology, Gokiso, Showa, Nagoya, Aichi 466-8555 Japan; 2 Elements Strategy Initiative for Catalysts and Batteries (ESICB), Kyoto University, 1–30 Goryo-Ohara, Nishikyo, Kyoto 615-8245 Japan; 3 Frontier Research Institute for Materials Science (FRIMS), Nagoya Institute of Technology, Gokiso, Showa, Nagoya, Aichi 466-8555 Japan; 4 Center for Materials research by Information Integration (CMI2), Research and Services Division of Materials Data and Integrated System (MaDIS), National Institute for Materials Science (NIMS), 1-2-1 Sengen, Tsukuba, Ibaraki, 305-0047 Japan; 5 Global Research Center for Environment and Energy based on Nanomaterials Science (GREEN), National Institute for Materials Science (NIMS), 1-1 Namiki, Tsukuba, Ibaraki, 305-0047 Japan; 6 Organization for Research Initiatives, Yamaguchi University, 2-16-1, Ube, 755-8611 Tokiwadai Japan; 7 Institute for Materials Chemistry and Engineering, Kyushu University, Kasuga koen 6-1, Kasuga, Fukuoka, 816-8580 Japan

## Abstract

Sodium ion batteries meet the demand for large-scale energy storage, such as in electric vehicles, due to the material abundance of sodium. In this report, nanotube-type Na_2_V_3_O_7_ is proposed as a cathode material because of its fast sodium diffusivity, an important requirement for sodium ion batteries, through the investigation of ~4300 candidates via a high-throughput computation. High-rate performance was confirmed, showing ~65% capacity retention at a current density of 10C at room temperature, despite the large particle size of >5 μm. A good cycle performance of ca. 94% in capacity retention after 50 cycles was obtained owing to a small volumetric change of <0.4%.

## Introduction

Sodium ion batteries (SIBs) are attractive alternatives for lithium ion batteries, which are currently preferred as power sources for environment-friendly large-scale batteries^1–3^. Sodium has a high ionization tendency comparable to that of lithium, which leads to high energy densities of SIBs^4^. Moreover, sodium resources are not limited; they are abundant in the earth’s crust as well as in seawater, ensuring a stable supply of SIBs to meet the increasing need for large-scale batteries^5^. Another expected advantage of SIBs is their higher sodium diffusivity compared to that of lithium due to weak interactions between alkaline ions and counter anions^4^. Thus, high rate performance— which is a critical requirement for electric vehicle applications^6^—is expected with appropriate crystal structure engineering.

**Figure 1:**
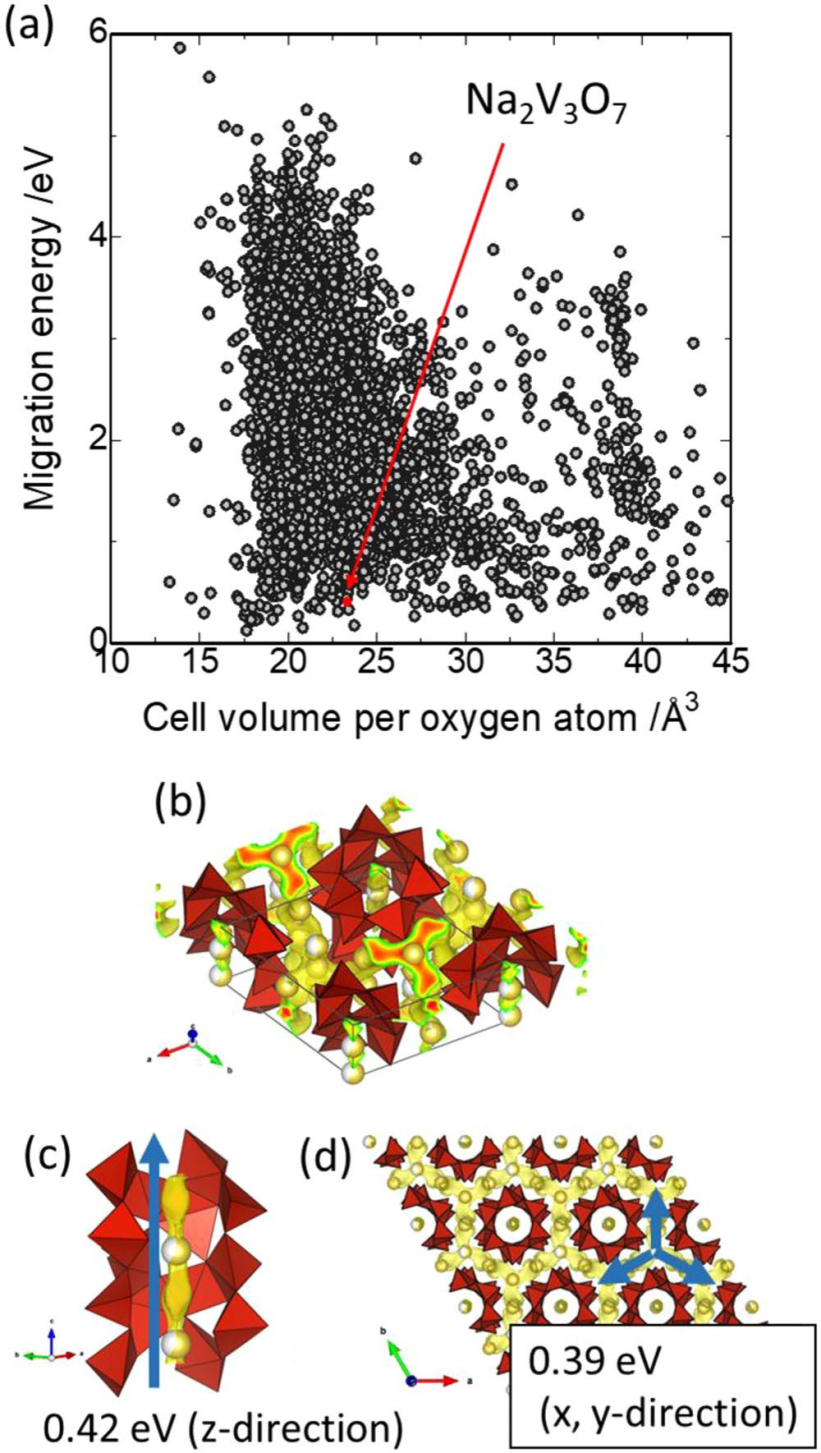
A total of 9096 inorganic solid-state samples that contained both Na and O were extracted from the inorganic crystal structure dataset (ICSD)^23^, and migration energies were evaluated for 4314 samples (gray symbols) using bond valence-based force field (BVFF)^24^ potential calculations and the percolation algorithm, as shown in panel (a)^7^. The rest of the materials showed high migration energies (>6 eV) or errors arising from unreasonable structure inputs, lack of a potential parameter set, spurious evaluation of oxidation states, etc. The red symbol in panel (a) corresponds to the Na_2_V_3_O_7_ compound with a nanotube structure (ICSD #88780). Panels (b–d) show the visualization of the isosurface with various potential energies obtained from BVFF calculations for Na_2_V_3_O_7_. The red polyhedra and yellow spheres represent VO_5_ and Na ions, respectively. The blue arrow in panel (c) corresponds to the Na ion migration pathway within the nanotube tunnel, and the calculated migration energy that percolated in the z-direction was 0.42 eV. The blue arrows in panel (d) represent the Na migration pathway at the peripheries of the nanotubes, and the calculated migration energy was 0.39 eV in the x and y directions. Note that the migration energy was defined as the difference between the maximum and minimum of the potential in the percolated pathway.

In this paper, the Na_2_V_3_O_7_ compound is suggested as a cathode material for SIB. This compound was chosen due to its expected high sodium diffusivity, predicted through high-throughput computations of sodium migration energies among 4314 compounds using the bond valence force field (BVFF) approach^7^ (Fig. 1(a)). Additionally, its sufficient theoretical capacity (~173 mAh g^−1^) and novelty (there have been few reports on its electrochemical performance, as mentioned later) are attractive. This compound was synthesized by P. Millet *et al*.^8^. One of the crystal structure features of Na_2_V_3_O_7_ is the formation of one-dimensional nanotubes consisting of VO_5_ square-pyramidal networks. These nanotubes are aligned as honeycomb structures, and the sodium ions are distributed inside and on the peripheries of the nanotubes. Therefore, the sodium ions inside and on the peripheries of the tubes diffuse one- and three-dimensionally, respectively, and the high-throughput computations predicted small migration energies for both routes (Fig. 1(b–d)). The nominal charge of V is +4 and thus a high voltage V^4+/5+^ redox reaction is expected accompanying sodium removal and insertion through intercalation. Hereinafter, we show the electrochemical performance and the crystal and electronic structure analyses of Na_2−x_V_3_O_7_ compounds as cathode materials for SIBs. Very recently, electrochemical measurements for Na_2_V_3_O_7_ cathode materials have been reported by Adamczyk *et al*. and in our previous work, confirming their rechargeable charge-discharge properties^9,10^. In this report, we present high rate and stable cycle performances, and the origin of these electrochemical performances is discussed thorough experimental and computational structure analyses.

## Results

### Structure of synthesized Na_2_V_3_O_7_ powder

**Figure 2:**
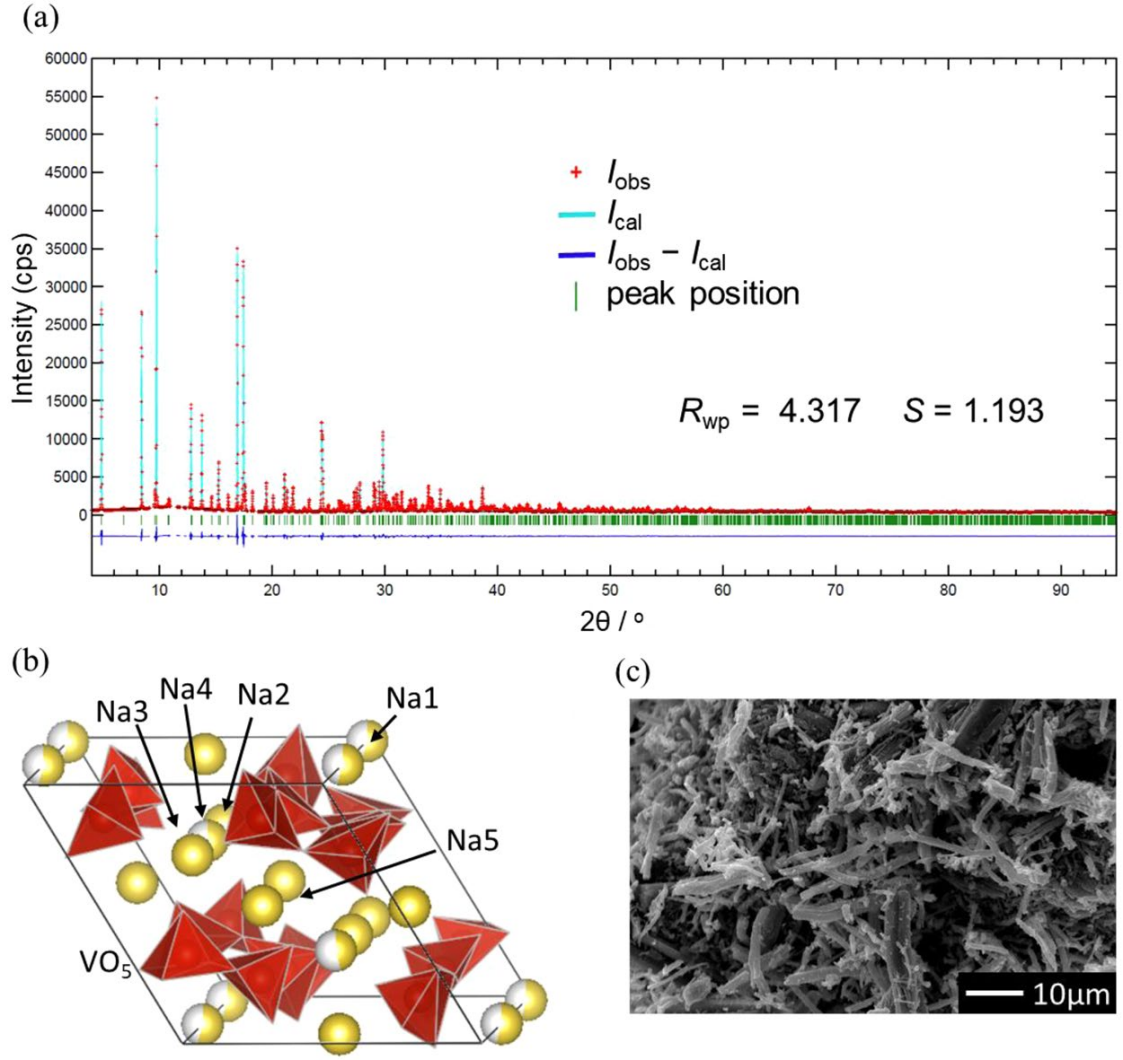
(**a**) Rietveld refinement XRD pattern for the synthesized Na_2_V_3_O_7_ particles. The adapted crystal structure model is shown in panel (b). A SEM image of the Na_2_V_3_O_7_ particles is displayed in panel (c).

Figure 2(a) and Supporting Table S1 show the Rietveld analysis results for the synchrotron X-ray diffraction (XRD) pattern of the synthesized Na_2_V_3_O_7_ powder. We confirmed the formation of a nanotube-type structure, as reported in a previous paper^8^ (Fig. 2(b)). Note that the weighted-profile *R*-factor (*R*_wp_) was improved by adding one sodium site (Na4 site) at the periphery of the nanotubes to the previous model^8^ (See details in Supporting Tables 1), where the presence of the Na4 site is indicated by a first-principles molecular dynamics (FPMD) simulation mentioned later in Supporting Section S2 and in Supporting Figure S5. By introducing Na4 sites, sodium ion occupancy of the Na1 site (inside nanotube) decreased from unity to ~0.5 in this study. Figure 2(c) shows a scanning electron microscope (SEM) image of the synthesized Na_2_V_3_O_7_. The particles had needle-shaped bodies with short-side sizes of *ca*. 5 µm and long-side sizes of *ca*. 30 µm. These sizes were one order of magnitude larger than the conventional sizes of the electrode particles for SIBs^11–13^. A transmission electron microscope (TEM) image and its selected area electron-diffraction pattern of the needle-like Na_2_V_3_O_7_ powder show that needle growth direction corresponds to the c-axis of Na_2_V_3_O_7_ (Supporting Figure S1). Because of the much longer diffusion distance along the c-axis, Na diffusion on the ab plane plays an important role.

### Characterization of Na_2_V_3_O_7_ electrodes

**Figure 3:**
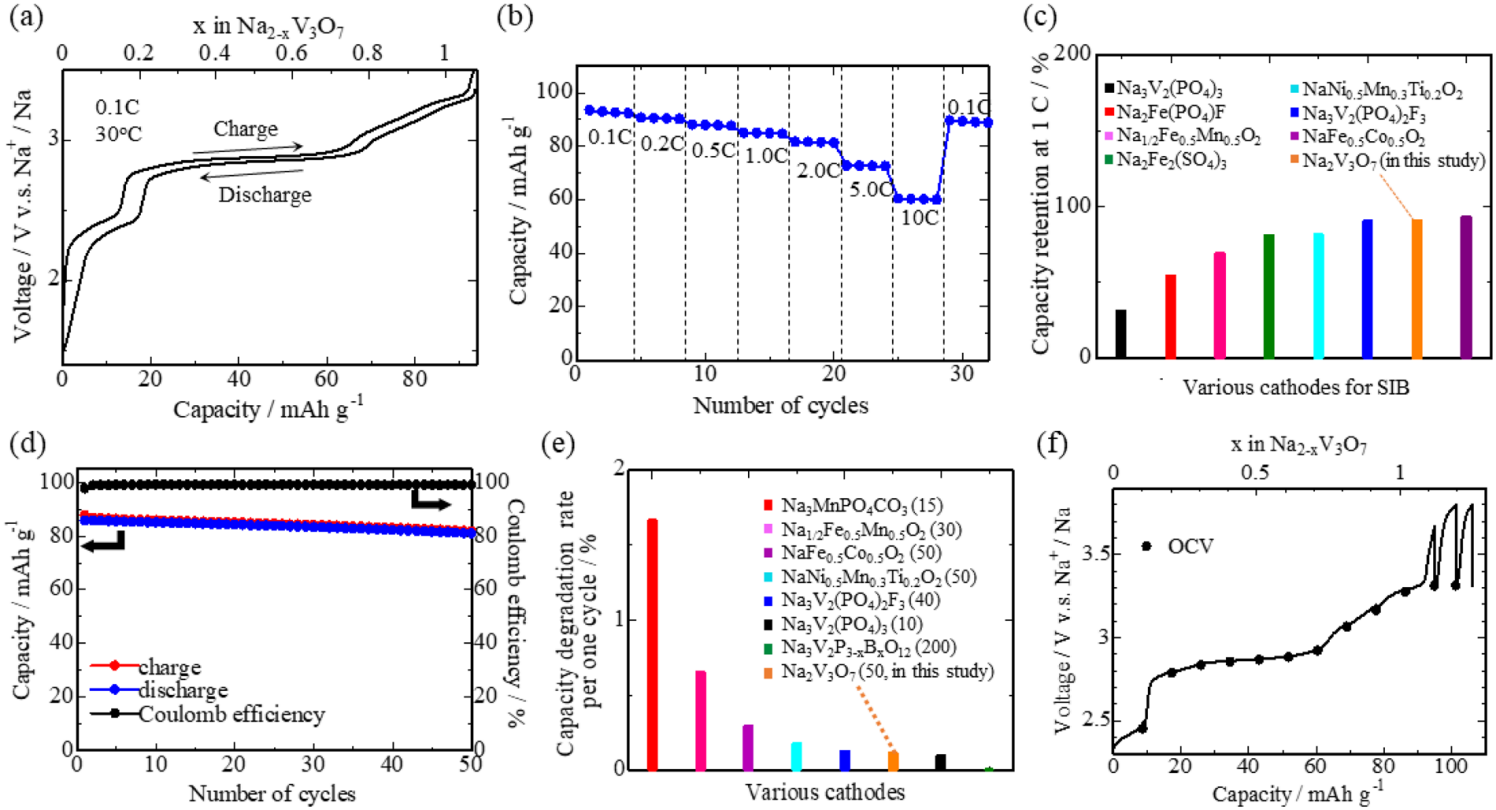
(**a**) The 1^st^ charge/discharge curves of a SIB using the Na_2_V_3_O_7_ electrode at a current density of 0.1C at 30 °C. The rate performance of the cell is shown in panel (b), whose capacity retention at 1C (against initial capacity at each lowest C-rate) is compared with those of other representative cathodes for SIBs^11–13,15–18^ in panel (c). The cycle performance of the cell at a current density of 1C is shown in panel (d). Capacity degradation rate per cycle is compared with those of other representative cathodes for SIBs^11,12,15–17,19,20^ in panel (e). Numbers in the circle brackets indicate the cycle number to assess the degradation rate. The galvanostatic intermittent titration technique (GITT) curve of the cell is shown in panel (f).

**Figure 4:**
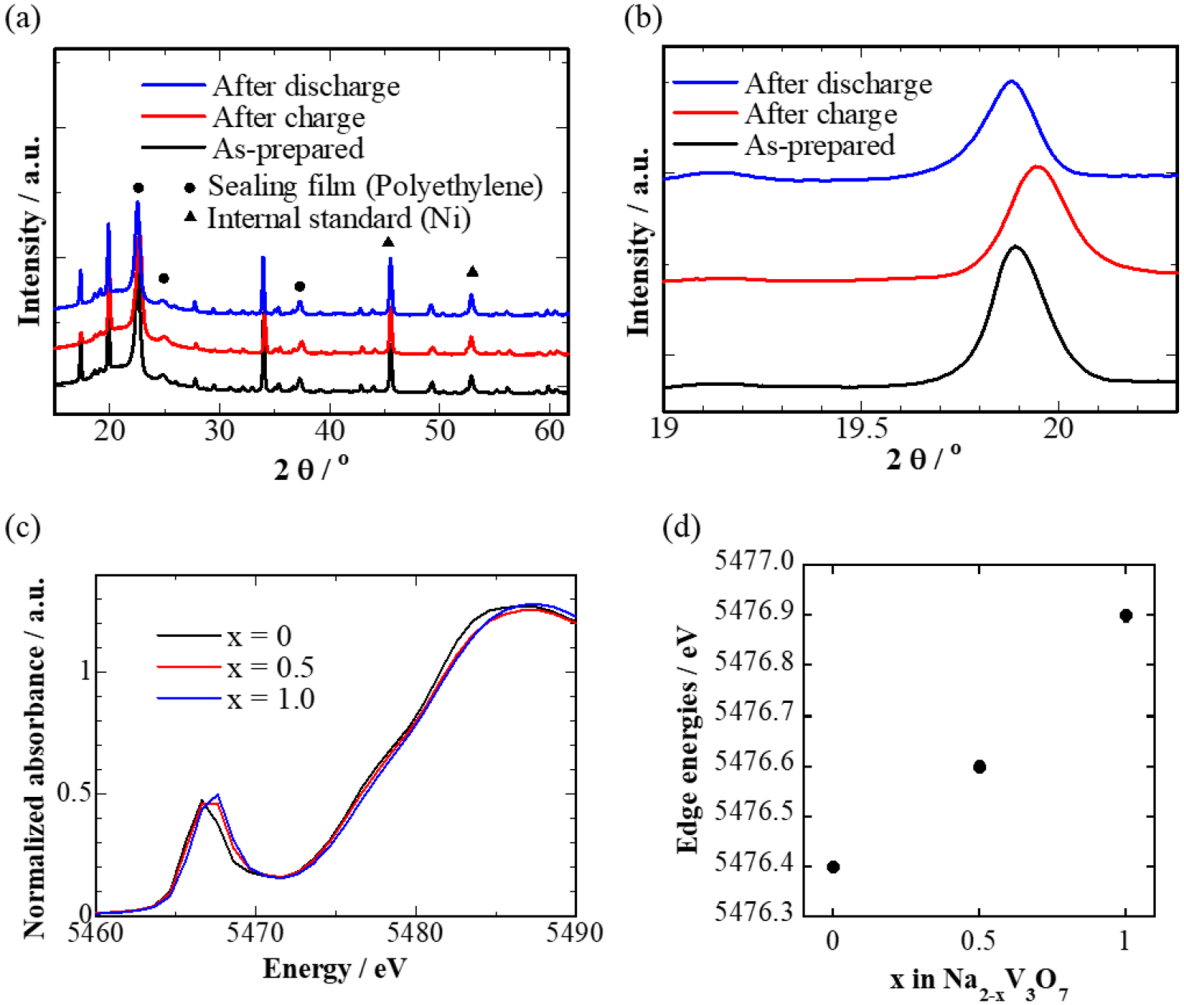
(**a**) XRD patterns of the Na_2_V_3_O_7_ electrode as-prepared and after charge and discharge processes. For clarity, the peak of Na_2_V_3_O_7_ with the strongest intensity is enlarged in panel (b). Normalized X-ray absorption near-edge spectroscopy (XANES) spectra at the V K-edges of Na_2−x_V_3_O_7_ electrode with different charge states of x = 0, 0.5, and 1. The edge energies at the normalized absorbance of 0.5 at the fronts of the peaks are compared in the spectra of Na_2−x_V_3_O_7_ in panel (d).

**Figure 5:**
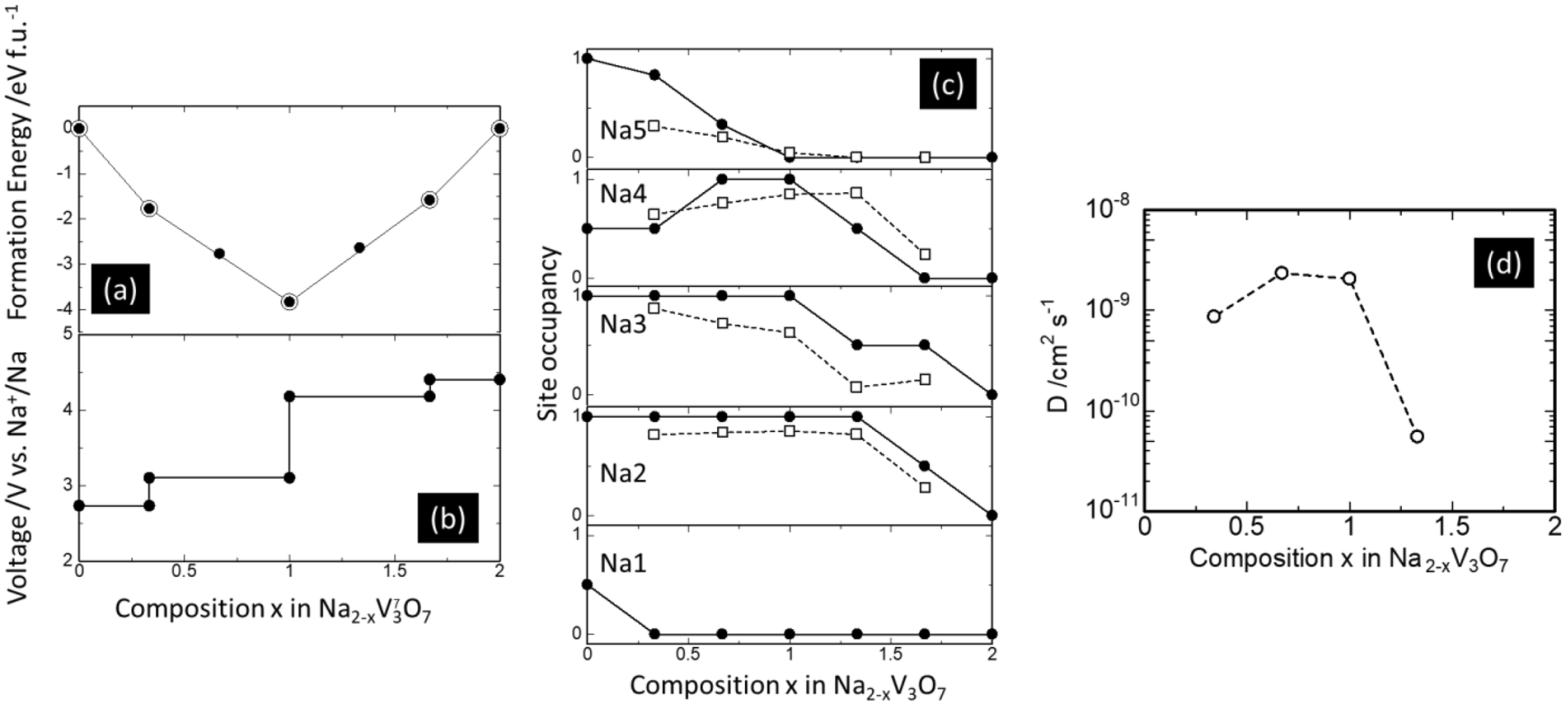
The optimization of Na/vacancy arrangement for Na_2−x_V_3_O_7_ (x = 0, 1/3, 2/3, 1, 4/3, 5/3, and 2) using genetic algorithm (GA). Panel (a) displays formation energy as a function of composition x in Na_2−x_V_3_O_7_ (solid circles). The open symbols and tie lines indicate the thermodynamic reaction path according to a convex hull. The solid circles and lines in panels (b and c) show the calculated voltage profiles and variations in Na occupancy for Na1–Na5 sites, respectively, versus composition x. The open symbols and hatched line in panel (c) correspond to molecular dynamics (MD) driven site occupancies. Panel (d) shows the molecular-dynamics-derived diffusion coefficient of Na at 1073 K as a function of composition x in Na_2−x_V_3_O_7_.

Figure 3(a) shows charge-discharge curves of the cell at a constant current density of 0.1C at 30 °C (C-rate is defined as the current to discharge the theoretical capacity in 1 hour: 1C) The Na_2_V_3_O_7_ electrode exhibited a reversible capacity of about 90 mAh g^−1^, and a sharp voltage increase followed when a voltage cutoff condition of 3.5 V vs. Li^+^/Li was reached. This voltage increase was attributed to Na/vacancy ordering at the composition x = 1 in Na_2−x_V_3_O_7_, as mentioned later. Indeed, the observed capacity corresponded to almost half of the theoretical capacity, ~173 mAh g^−1^, for 2 mol desodiation from the Na_2_V_3_O_7_ compound. We confirmed that the structural topology was roughly unchanged from the XRD profiles during sodium extraction (Fig. 4(a,b)) and a gradual edge-energy increase in the V K-edge X-ray absorption near edge spectra (XANES) (Fig. 4(c,d)), indicating a reversible sodium intercalation reaction process accompanying V redox (V^4+/5+^). These were supported by first-principles DFT calculations with genetic algorithm (GA) optimization of the sodium/vacancy arrangement (see details in Supporting Section S1). Figure 5 shows (a) the calculated formation energies for Na_2−x_V_3_O_7_ in which the total electron energies for the end compositions (x = 0 and 2) were set to zero, (b) the calculated voltage profile, and (c) occupancies of sodium ions for Na1–Na5 sites as functions of composition x in Na_2−x_V_3_O_7_. The calculated voltage curves also agree with the experimental results (see Fig. 3(a)). During the early stages of desodiation (0 ≤ x ≤ 1/6), the sodium ions inside the nanotube (Na1 site) were removed due to instability and thus the voltages (2.7 V) were lower than the following desodiation process (>3.0 V for 1/3 ≤ x ≤ 1) in which sodium ions were removed from nanotube-fringe sites (Na2-Na5 sites). After desodiation from tunnel sites, electrochemical sodium removal from nanotube-fringe sites showed two voltage steps at ~3.0 V and >4.0 V before and after desodiation to x = 1, respectively. Since there were no significant changes in the V_3_O_7_ framework and the redox mechanism of V ions (3+/4+) at composition x = 1.0, we inferred that the calculated voltage change was due to ordering of the Na/vacancy arrangement. Indeed, the ordered arrangement was indicated in the GA optimization at x = 1, where all the Na5 sites were vacant, while the rest of the nanotube-fringe sites (Na2–Na4) were occupied by sodium ions. This was confirmed by first-principles molecular dynamics (FPMD) simulations, mentioned later in Supporting Section S2. The averaged Bader charges^14^ of V ions shown in Supporting Figure S4 showed a monotonic increase, whereas no marked changes occurred on the Na and O ions (not shown). Thus, vanadium ions were responsible for the redox reaction of the charge/discharge reaction, which agreed with the XANES spectra shown in Fig. 4(c,d).

Figure 3(b) shows galvanostatic discharge capacities with various current densities (using units of C-rate). The C-rate increased gradually every 4 cycles from 0.1 C to 10 C, and then 0.1 C cycling was repeated. Remarkably good capacity retention of ~65% (against the initial capacity at 0.1 C) was obtained even at 10 C, corresponding to full discharge within 6 minutes. Good rate performance was confirmed through comparison with other representative cathodes for SIBs^11–13,15–18^, as shown in Fig. 3(c). Figure 3(d) shows the cycle performance of the Na_2_V_3_O_7_ electrode at a current density of 1 C. The Na_2_V_3_O_7_ electrode also exhibited excellent cycle performance in which the capacity retentions were *ca*. 94% at the 50^th^ cycle. The coulombic efficiency was almost 100% up to 50 cycles. This was confirmed by comparison of capacity degradation with those of other representative cathodes^11,12,15–17,19,20^, as shown in Fig. 3(e), which indicated a small capacity fade per cycle for Na_2_V_3_O_7_. The good cycle performance arises from the small volumetric change in the V_3_O_7_ host lattice, *ca*. −3.2 vol% at 3.8 V, which was calculated using the lattice parameter change obtained from *ex-situ* XRD profiles (Supporting Table S2). A SEM image of the Na_2_V_3_O_7_ electrode after the 50 charge-discharge cycles also indicated that the tube structures of the as-prepared Na_2_V_3_O_7_ particles remained (Supporting Figure S3). Note that the present results show much better rate and cycle performances than the recent work by Adamczyk *et al*.^9^. We inferred that the difference stems from (1) the higher charge cutoff voltage (4.5 V) than the present condition (3.5 V), which causes slow Na^+^ diffusion, as mentioned later, and (2) inclusion of the impurity phase, Na_9_V_14_O_35_ (7%), in the recent report^9^.

As mentioned above, the observed reversible capacity was half of the theoretical value at the upper cutoff voltage of 3.5 V. The galvanostatic intermittent titration technique (GITT) was used to obtain the open circuit voltage and polarization (overvoltage) of the highly desodiated region, x > 1 in Na_2−x_V_3_O_7_, as shown in Fig. 3(f). Further charging was confirmed for x > ~1 accompanying large polarization, indicating that slow kinetics regulate the capacity. Figure 5(d) displays the variation of the MD-derived diffusion coefficients of Na versus composition x (0.33 ≤ x ≤ 1.33) at a temperature of 1073 K (see details in Supporting Section S2). A significant decrease in the sodium diffusion coefficient was indicated at x > ~1 in Na_2−x_V_3_O_7_ where the Na5 sites were almost empty, which agrees with the results of the AC impedance measurements (Supporting Section S3 and Supporting Figure S6). The sharp drop in sodium diffusivity at x > ~1 was ascribed to Na/vacancy ordering from the computational results of the GA-derived structure and FPMD-averaged structures (Fig. 5(c)), which prevented further desodiation.

In summary, the Na_2_V_3_O_7_ compound, which was selected among ~4300 candidates due to fast sodium diffusivity via a high-throughput computation, was evaluated as a cathode material for SIBs. This compound showed excellent rate performance and cyclability, though the capacity was limited to half of the theoretical capacity (~173 mAh g^−1^). Since the ordering of Na/vacancy prevented further sodium extraction, doping with appropriate cations or anions would disturb the orderings and improve the capacity. The observed good rate and cycle performances were confirmed for a large particle size of ~5 μm (Fig. 2(c)). Thus, it is expected that nanoparticle synthesis of this compound will largely improve the electrochemical performance of SIBs.

## Methods

### Sample preparation

The Na_2_V_3_O_7_ material was synthesized by a solid-state reaction method based on a previous report^8^. First, Na_2_CO_3_ (purity 99.8%, Wako) and V_2_O_5_ (99.0%, Wako) powders were mixed in a 4:3 molar ratio. The powder was pelletized and then heated at 650 °C for 1 h to prepare precursor Na_4_V_3_O_7_. The Na_4_V_3_O_7_ and V_2_O_5_ mixture and V_2_O_3_ (99.0%, Kojyundo Kagaku) powder were mixed in an Ar-filled glove box to yield a chemical composition of Na_2_V_3_O_7_. The mixture pellet was covered with Au foil and sealed under a vacuum in a silica tube. Na_2_V_3_O_7_ was prepared by sintering the pellet at 700 °C for 24 h. All sample manipulations were conducted under an inert gas atmosphere, unless specifically mentioned.

### Sample characterization

Powder X-ray diffraction experiments were conducted at BL5S2 of the Aichi Synchrotron Radiation Center, Japan. The sample powders were packed in a 0.3 mm diameter borosilicate glass capillary tube and sealed with an epoxy plastic in an Ar-filled glove box. Diffraction measurements were performed in transmission geometry using X-rays with 0.8 Å wavelengths. Diffraction data were collected in 0.01° steps from 4° to 94.91° in 2θ with a scan rate of 2.37° min^−1^. The computer program RIETAN-FP^21^ was used for the Rietveld analysis. The synthesized Na_2_V_3_O_7_ particles were observed using a scanning electron microscope (SEM; JEOL, JSM-6360LV). The accelerating voltage was 20 kV.

### Electrochemical characterization

Electrochemical performances were investigated for 2032-type coin cells using charge-discharge equipment (VMP3, Bio-Logic). Composite cathodes were prepared by mixing the Na_2_V_3_O_7_ active material, Ketjenblack conductive additive, and polyvinylidene fluoride binder at an 8:1:1 weight ratio in *N*-methyl-2-pyrrolidone. The slurry was cast on an Al foil with an average loading of *ca*. 1.2 mg cm^−2^ and dried at 110 °C overnight in a vacuum prior to use. Propylene carbonate with 1 M NaClO_4_ and 2 wt% fluoroethylene carbonate was used as the electrolyte solution. The cells were assembled with Na anodes and cycled galvanostatically at different current rates at 30 °C in the voltage range of 1.5–3.5 V. The Na reference electrode was also introduced to analyse cell resistances. The overvoltage was quantified by the galvanostatic intermittent titration technique (GITT). The cells were charged for 1 h at a current density of 0.05 C and relaxed for 5 h. The relaxation time was changed to 10 h for voltages over 3.3 V because the relaxation of overvoltage was slow. The resistance was separated and analysed by AC impedance measurements. The applied voltage and measurement frequencies were 10 mV and 1 mHz–0.5 MHz.

### Structure analysis for Na_2−x_V_3_O_7_ electrodes

XANES measurements for the charged Na_2−x_V_3_O_7_ electrodes were carried out at the BL5S1 beam line of the Aichi Synchrotron Radiation Center, Japan. The electrodes were prepared by charging to correspond to the compositions, washing with propylene carbonate, and drying at 70 °C overnight. The V K-edge spectra were obtained in transmission mode at room temperature. XRD measurements for the electrode after the charge and discharge processes were conducted with Ni powders as internal standards for peak positions. The electrodes were washed with propylene carbonate and dried at 70 °C overnight. These samples were sealed with polyethylene films and measured by an X-ray diffractometer (Mini Flex 600, Rigaku) using Cu-K*α* radiation. Crystal structure diagrams were drawn with Visualization for Electronical and Structural Analysis (VESTA)^22^.

## Electronic supplementary material

Supplementary Information
