# Supplementary material for: Nanotube-structured Na2V3O7 as a Cathode Material for Sodium-Ion Batteries with High-rate and Stable Cycle Performances
Source: Sci Rep. 2018 Nov 21;8:17199. doi: 10.1038/s41598-018-35608-9 (PMC6249327; doi:10.1038/s41598-018-35608-9)
Supplement: Supplementary file 1 — Supplementary Information [file 41598_2018_35608_MOESM1_ESM.doc]

**Supporting Information**

Nanotube-structured Na_2_V_3_O_7_ as a Cathode Material for Sodium-Ion Batteries with High-rate and Stable Cycle Performances

Naoto Tanibata^1,2*^, Yuki Kondo^1^, Shohei Yamada^1^, Masaki Maeda^1^, Hayami Takeda^1,2^, Masanobu Nakayama^1-5^, Toru Asaka^1,6^, Ayuko Kitajou^2,7,8^, Shigeto Okada^2,7^.

^1^ Department of Advanced Ceramics, Nagoya Institute of Technology, Gokiso, Showa, Nagoya, Aichi 466-8555, Japan

^2^ Elements Strategy Initiative for Catalysts and Batteries (ESICB), Kyoto University, 1-30 Goryo-Ohara, Nishikyo, Kyoto 615-8245, Japan

^3^ Frontier Research Institute for Materials Science (FRIMS), Nagoya Institute of Technology, Gokiso, Showa, Nagoya, Aichi 466-8555, Japan

^4^ Center for Materials research by Information Integration (CMI2), Research and Services Division of Materials Data and Integrated System (MaDIS), National Institute for Materials Science (NIMS), 1-2-1 Sengen, Tsukuba, Ibaraki 305-0047, Japan

^5^ Global Research Center for Environment and Energy based on Nanomaterials Science (GREEN), National Institute for Materials Science (NIMS), 1-1 Namiki, Tsukuba, Ibaraki 305-0047, Japan

6 Frontier Research Institute for Materials Science (FRIMS), Nagoya Institute of Technology, Gokiso-cho, Showa-ku, Nagoya, 466-8555, Japan

^7^ Institute for Materials Chemistry and Engineering, Kyushu University, Kasuga koen 6-1, Kasuga, Fukuoka 816-8580, Japan

^8^ Organization for Research Initiatives, Yamaguchi University, 2-16-1 Tokiwadai, Ube, 755-8611, Japan

*corresponding author: [tanibata.naoto@nitech.ac.jp](mailto:tanibata.naoto@nitech.ac.jp)

**Supporting Section S1: DFT+U study on phase stability for Na_2-x_V_3_O_7_.**

First-principles density functional theory (DFT) calculations were performed to investigate the phase stabilities of Na_2-x_V_3_O_7_ (x = 0, 1/3, 2/3, 1, 4/3, 5/3, and 2) using the Vienna *ab initio* simulation package (VASP)^1,2^ with the projector augmented-wave (PAW) method^3^ and plane-wave basis set. A generalized gradient approximation (GGA)-type exchange-correlation functional developed by Perdew, Burke, and Ernzernhof and modified for solid materials (PBEsol)^4,5^ was used. For crystal structure relaxation and evaluation of the total electron energy, an on-site Coulomb correction (GGA + U) was included to describe localized electronic states in V 3*d* orbitals. U was set to 4.2 eV for V 3*d* states based on a linear response approach^6–8^ using the Quantum-ESPRESSO software package.^9,10^

A genetic algorithm approach with DFT + U calculations was used to determine the most stable Na and vacancy arrangement in Na_2-x_V_3_O_7_. The crystal structure inputs, lattice parameters, and fractional coordinates of the ions were obtained from the present Rietveld analysis for synchrotron X-ray diffraction in the main text and first-principles molecular dynamics study, as mentioned later. Five crystallographic sites (Na1-Na5) listed in **Supporting Table S1 (b)** and 14 sites for Na ions and vacancies in a primitive cell of Na_2-x_V_3_O_7_ were considered in this study. The details of the GA approach are discussed elsewhere.^11,12^

The GA optimized structure for pristine Na_2_V_3_O_7_ indicated that all Na1 sites (nanotube tunnel sites) were vacancies, while the rest were fully occupied by Na ions. However, each half of the Na1 and Na4 sites was occupied by Na ions for the second-best structure, which agrees with the present Rietveld refinement. The energy difference between the first and second stable structures was ~80 meV/Na_2_V_3_O_7_. We inferred that the configurational entropy term stabilized the second-best structure in the present GA approach (our rough estimation of the phase transition from the best to second-best structure was above 350 K, obtained only by considering the configurational freedom of Na/vacancy arrangement). Thus, the second-best structure was adopted for Na_2_V_3_O_7_, hereinafter, unless specifically mentioned.

**Supporting Section S2: DFT-based molecular dynamics study on sodium ion diffusion in Na_2-x_V_3_O_7_ compounds**

We performed DFT-based first-principles molecular dynamics (FPMD) simulations using VASP to investigate the diffusion of Na in Na_2-x_V_3_O_7_ (x = 1/3, 2/3, 1, and 4/3) systems (total 248–280 atoms in the simulation cell). Note that the Na1 sites (inside the nanotube) were empty for the calculated compositions. The cutoff energy was set to 350 eV and a 1 × 1 × 1 *k*-point grid (only Γ point) was employed to reduce the computational cost. The time step was set to 1 fs and our FPMD simulations were carried out in a canonical ensemble (NVT ensemble) using a Nosé thermostat^30^ at 1073 K for > 50 ps. Note that, though the experimental electrochemical tests were carried out at room temperature, the temperature for the FPMD simulations was rather high. This is due to the limit of computational resources, where the number of Na ions in the cell and simulation times were not large enough to accumulate Na hopping events to a statistically sufficient level. From our FPMD simulations, the time-average mean square displacements (MSD) of all elements were calculated, as displayed in **Supporting Figure 4(a)** at the composition x = 1 in Na_2-x_V_3_O_7_. We confirmed that the Na ion solely diffused in the lattice, while V and O ions remained near their original sites (i.e., thermal vibration). The corresponding Arrhenius plot and calculated diffusion coefficients are shown in panel (b). The estimated migration energy of the Na ions was 0.45 eV, showing reasonable agreement with the BVFF-derived migration energy (**Figure 1** in the main text).

Trajectories of Na ion migration in the lattice are shown in **Supporting Figure S5**, showing good agreement with the BVFF-calculation-derived trajectories. Na occupancy was confirmed at Na2-Na5 in panels (b) and (d) at the nanotube-fringe space. Na occupancy at Na4 sites was predicted, which has not been reported so far. Thus, we introduced Na4 sites for the present Rietveld analysis for synchrotron XRD (**Figure 2(a)** and **Supporting Table S1**), and GA modelling (**Supporting Section S1**). The three-dimensional network of Na ion migration was clearly visible among the nanotube-fringe sites (Na2-Na5). In detail, the Na2-Na3, Na3-Na5, Na5-Na4, and Na5-Na5 sites were connected, forming zigzag pathways along the c-axis. We also calculated site occupancies by spherical integration of the probability density within a radius of 1.2 Å from Na2-N4 sites; the corresponding results are plotted as open symbols (hatched line) in **Figure 5(c)** in the main text. It was confirmed that the MD-derived site occupancies agreed with the GA-derived result. Therefore, the present MD calculations also predicted the formation of Na/vacancy ordering at a composition x ≈ 1.

**Supporting Section S3: AC impedance study**

**Supporting Figure 4(a)** depicts the voltage-time relationship for the galvanostatic intermittent titration technique shown in **Figure 3(f)** in the main text. The magnified relaxation curves at compositions x ≈ 0.3 and x ≈ 1.0 are shown in panels (b) and (c) in the figure, respectively. The voltage dropped sharply as a function of time in the relaxation process, as shown in panel (b), indicating that the polarization was mainly due to resistance of the electrolyte solution and/or a charge transfer process at x < ~1.0. However, a gradual decrease in the voltage during relaxation was visible at x > ~ 1.0, indicating that the reaction kinetics was controlled by the diffusion process. Panels (d) and (e) of the figure display the AC impedance spectra, showing two semicircles in the higher frequency region and a Warburg-type straight line in the lower frequency region. The summation of semicircles, which might correspond to resistance of sodium transport in the electrolyte solution and/or SEI and a charge transfer process at the interface of the electrolyte | electrode^13^, increased from 120 to 280 Ω with compositions x from ~0.3 to ~1.0. However, the estimated polarizations of their resistances at a higher frequency at x = ~0.3 and ~1.0 were 2.16 and 5.30 mV, respectively, which corresponded to 54% and 1.5% of the polarization observed in the GITT relaxation measurements (~5 mV and ~ 350 mV). Accordingly, an abrupt increase in GITT-derived polarization at x > ~1.0 was attributed to the diffusion process, i.e., impedance observed in the lower frequency region, as mentioned above.

**References**

1. Kresse, G. & Furthmüller, J. Efficient iterative schemes for *ab initio* total-energy calculations using a plane-wave basis set. *Phys. Rev. B* **54,** 11169 (1996).

2. Kresse, G. & Furthmiiller, J. Efficiency of ab-initio total energy calculations for metals and semiconductors using a plane-wave basis set. *Comput. Mater. Sci.* **6,** 15–50 (1996).

3. Blöchl, P. E. Projector augmented-wave method. *Phys. Rev. B* **50,** 17953 (1994).

4. Perdew, J. P., Burke, K. & Ernzerhof, M. Generalized gradient approximation made simple. *Phys. Rev. Lett.* **77,** 3865 (1996).

5. Perdew, J. P. *et al.* Restoring the Density-gradient expansion for exchange in solids and surfaces. *Phys. Rev. Lett.* **100,** 136406 (2008).

6. Cococcioni, M. & de Gironcoli, S. Linear response approach to the calculation of the effective interaction parameters in the LDA + U method. *Phys. Rev. B* **71,** 035105 (2005).

7. Kulik, H. J., Cococcioni, M., Scherlis, D. A. & Marzari, N. Density Functional Theory in Transition-Metal Chemistry: A Self-Consistent Hubbard U Approach. *Phys. Rev. Lett.* **97,** 103001 (2006).

8. Kulik, H. J. & Marzari, N. Systematic study of first-row transition-metal diatomic molecules: A self-consistent DFT+U approach. *J. Chem. Phys.* **133,** 114103 (2010).

9. Giannozzi, P. *et al.* QUANTUM ESPRESSO: a modular and open-source software project for quantum simulations of materials. *J. Phys. Condens. Matter* **21,** 395502 (2009).

10. Giannozzi, P. *et al.* Advanced capabilities for materials modelling with Quantum ESPRESSO. *J. Phys. Condens. Matter* **29,** 465901 (2017).

11. Yabuuchi, N. *et al.* Origin of stabilization and destabilization in solid-state redox reaction of oxide ions for lithium-ion batteries. *Nat. Commun.* **7,** 13814 (2016).

12. Itoh, T., Mori, M., Idemoto, Y., Imai, H. & Nakayama, M. Annealing effect on phase stability of doped zirconia using experimental and computational studies. *Solid State Ionics* **297,** 20–28 (2016).

13. Nakayama, M. *et al.* Combined computational and experimental study of Li exchange reaction at the surface of spinel LiMn_2_O_4_ as a rechargeable Li-ion battery cathode. *J. Phys. Chem. C* **118,** 27245–27251 (2014).

**Supporting Table S1** Structural parameters after Rietveld refinement using model (a) ICSD (w/o Na4 sites) and (b) FPMD-derived structure (w/ Na4 sites).


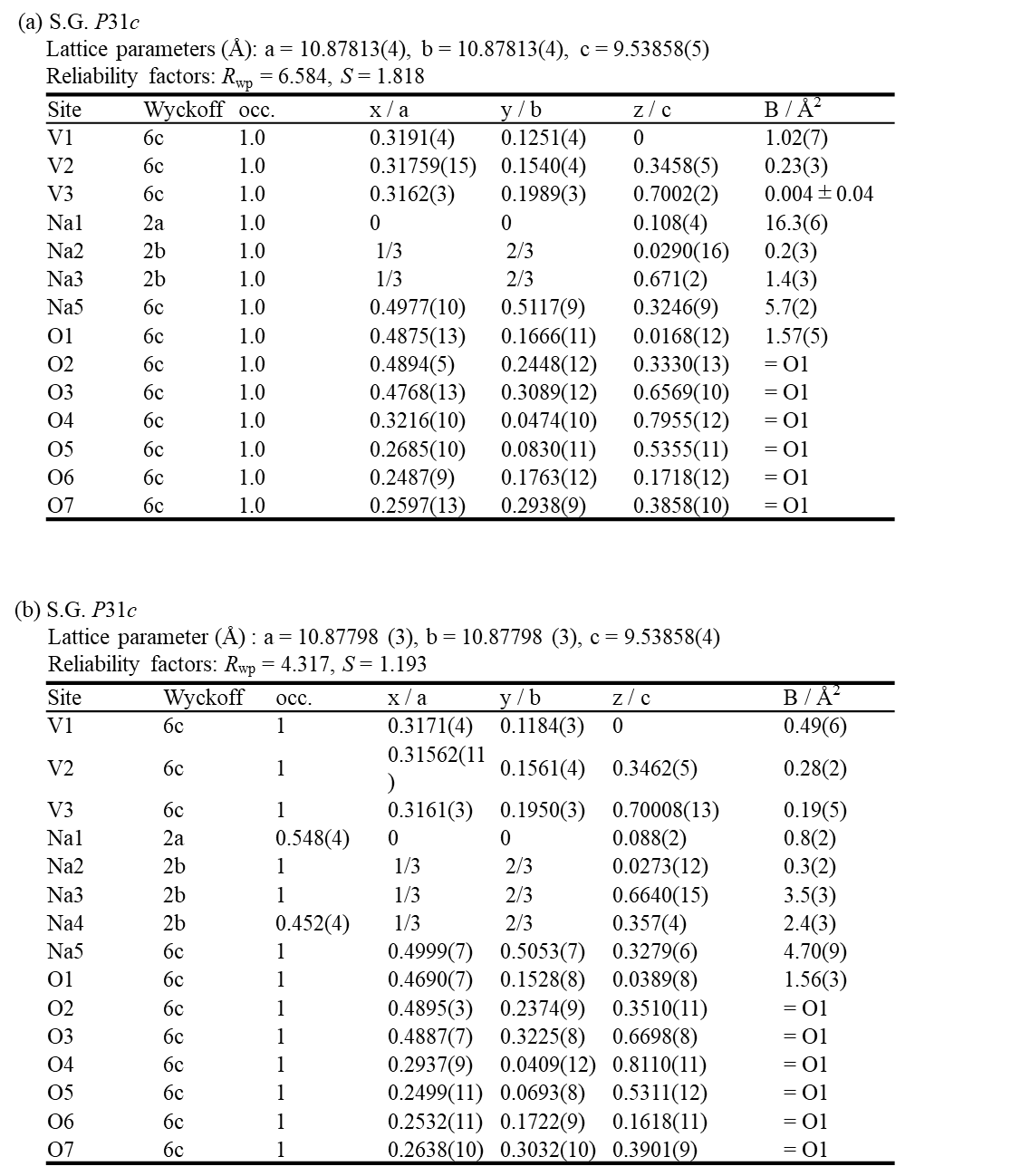


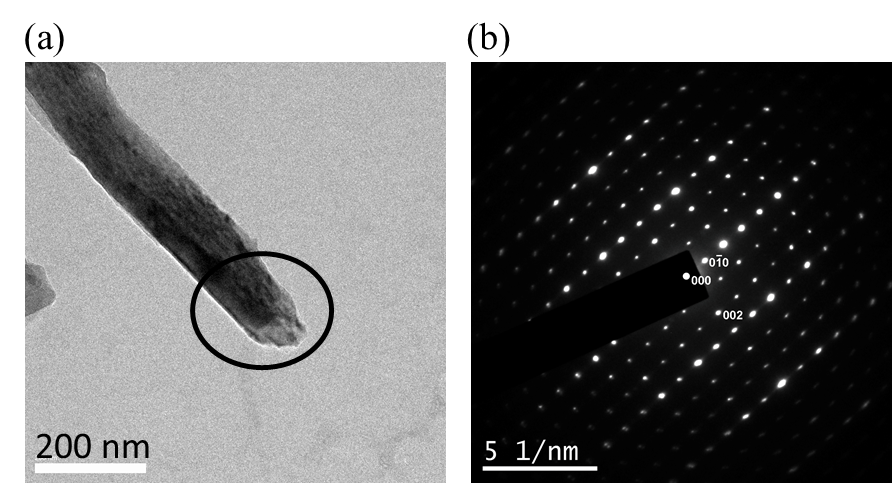


**Supporting Figure S1** (a) A bright-field image and (b) selected area electron-diffraction (SAED) patterns of the as-prepared Na_2_V_3_O_7_ powder by a transmission electron microscopy (TEM). The needle-shape morphology is shown. The SAED pattern was obtained from the open circle region in the bright-field image. The SAED pattern indicated that the needle growth direction corresponds to the c-axis of the nanotube-type Na_2_V_3_O_7_ crystal structure.


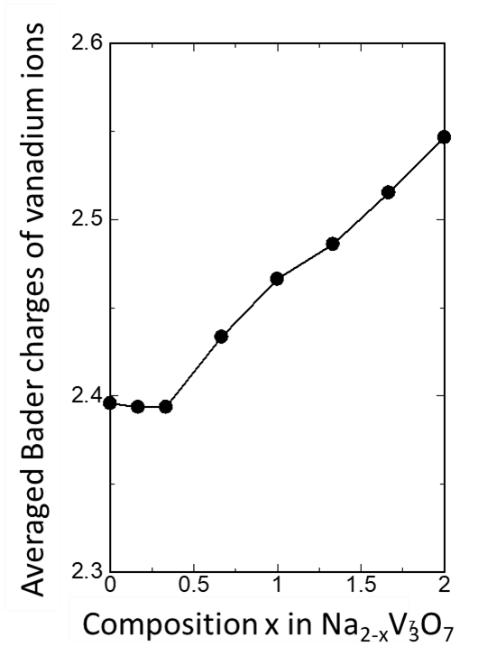


**Supporting Figure S2** Variation in average Bader charge of vanadium ions in the Na_2-x_V_3_O_7_ lattice from DFT-calculated electron densities (see details in **Supporting Section 1**).

**Supporting Table S2** Lattice parameters and cell volumes of Na_2_V_3_O_7_ active material before and after charge/discharge processes and their volume change percentages, which were calculated from the XRD patterns (shown in **Figure 4(a)** in the main text).

|  | a, b / Å | c / Å | Volume (V) / Å^3^ | ΔV / % |
| --- | --- | --- | --- | --- |
| Before charge | 9.2204 | 9.4943 | 932.036 | 0 |
| After charge | 9.2113 | 9.2078 | 902.121 | -3.2 |
| After discharge | 9.2363 | 9.4300 | 928.922 | -0.3 |


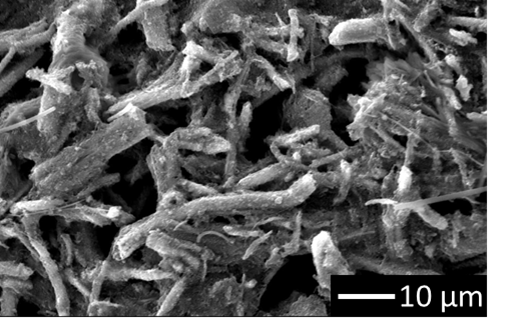


**Supporting Figure S3** SEM image of the Na_2_V_3_O_7_ particles in the sodium-battery electrode after 50 charge-discharge cycles.


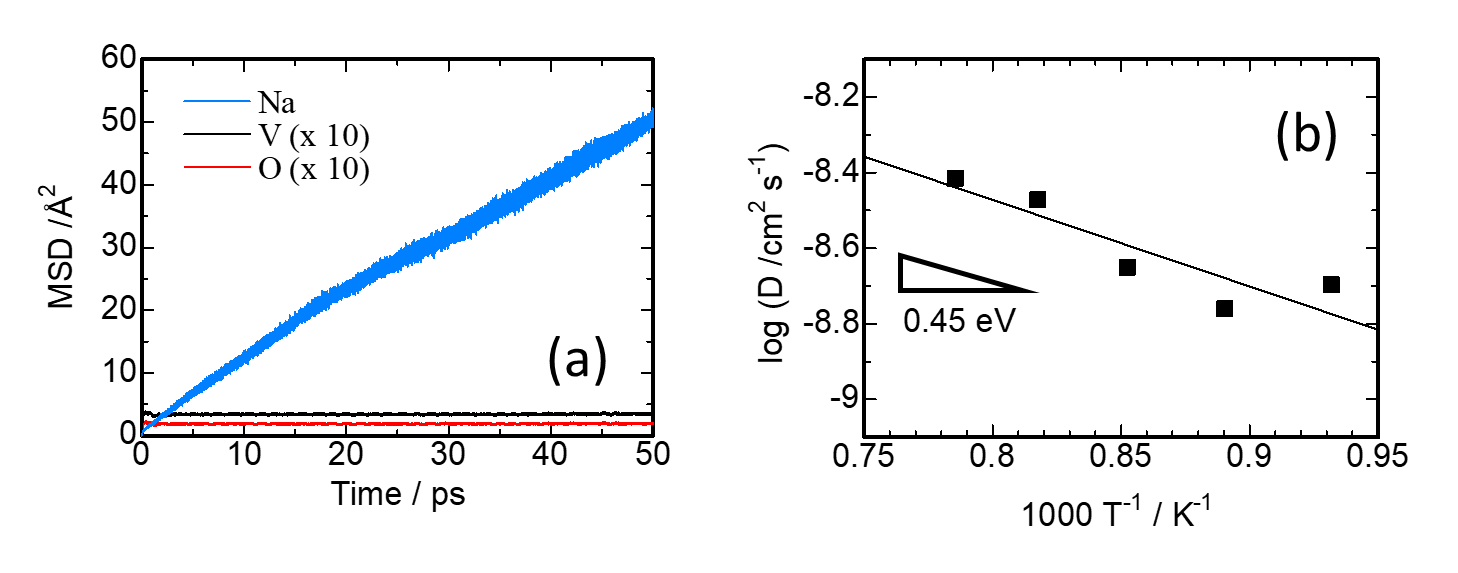


**Supporting Figure S4** (a) MSD plots of Na, V, and O trajectories in NaV_3_O_7_ (x = 1) at 1173 K. Plots for V and O are magnified by a factor of 10 to illustrate their behaviours. (b) Arrhenius plot of temperature-dependent sodium ion diffusion coefficient with linear fit line. The calculated activation energy (migration energy) was 0.45 eV.


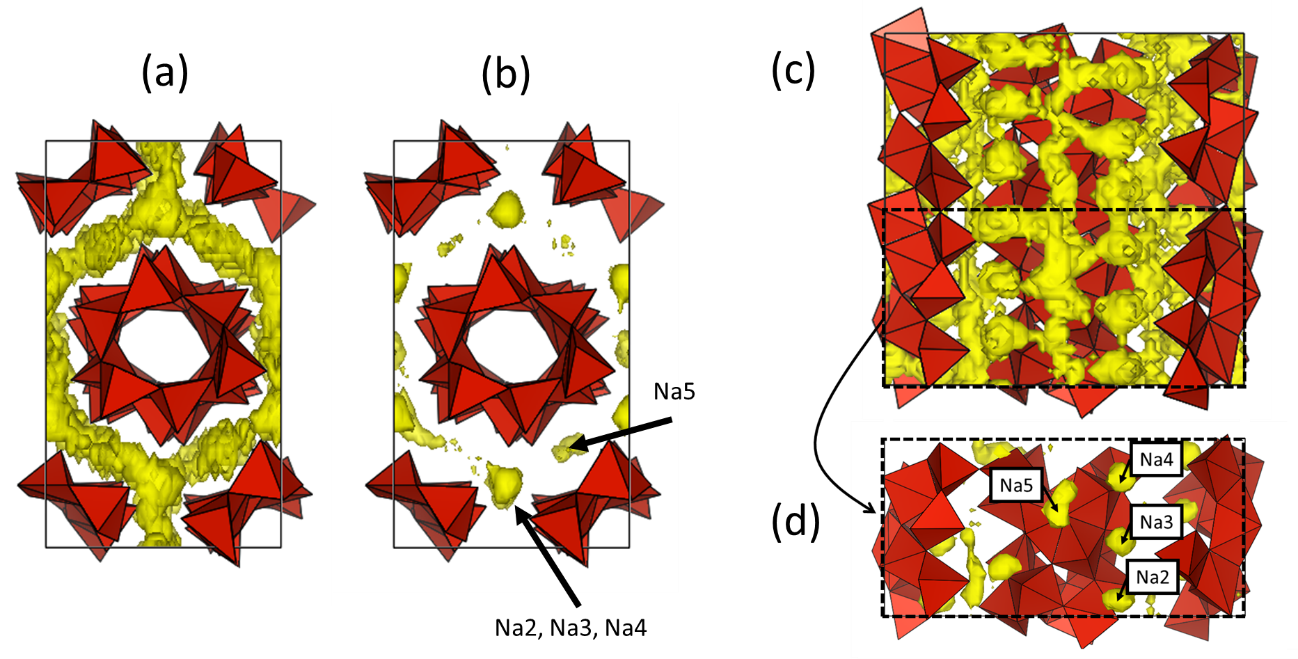


**Supporting Figure S5** Na^+^ probability density distribution in NaV_3_O_7_ (x = 1.0) taken from 30 ps MD simulation run (post-equilibration). The red polyhedra represent VO_5_. Panels (a) and (b) correspond to the projection view along the [001] direction, and (c) and (d) are along the [100] direction. The isosurface level was set to 2 × 10^-3^ Å^-3^ for panels (a) and (c) and 0.1 Å^-3^ for (b) and (d).


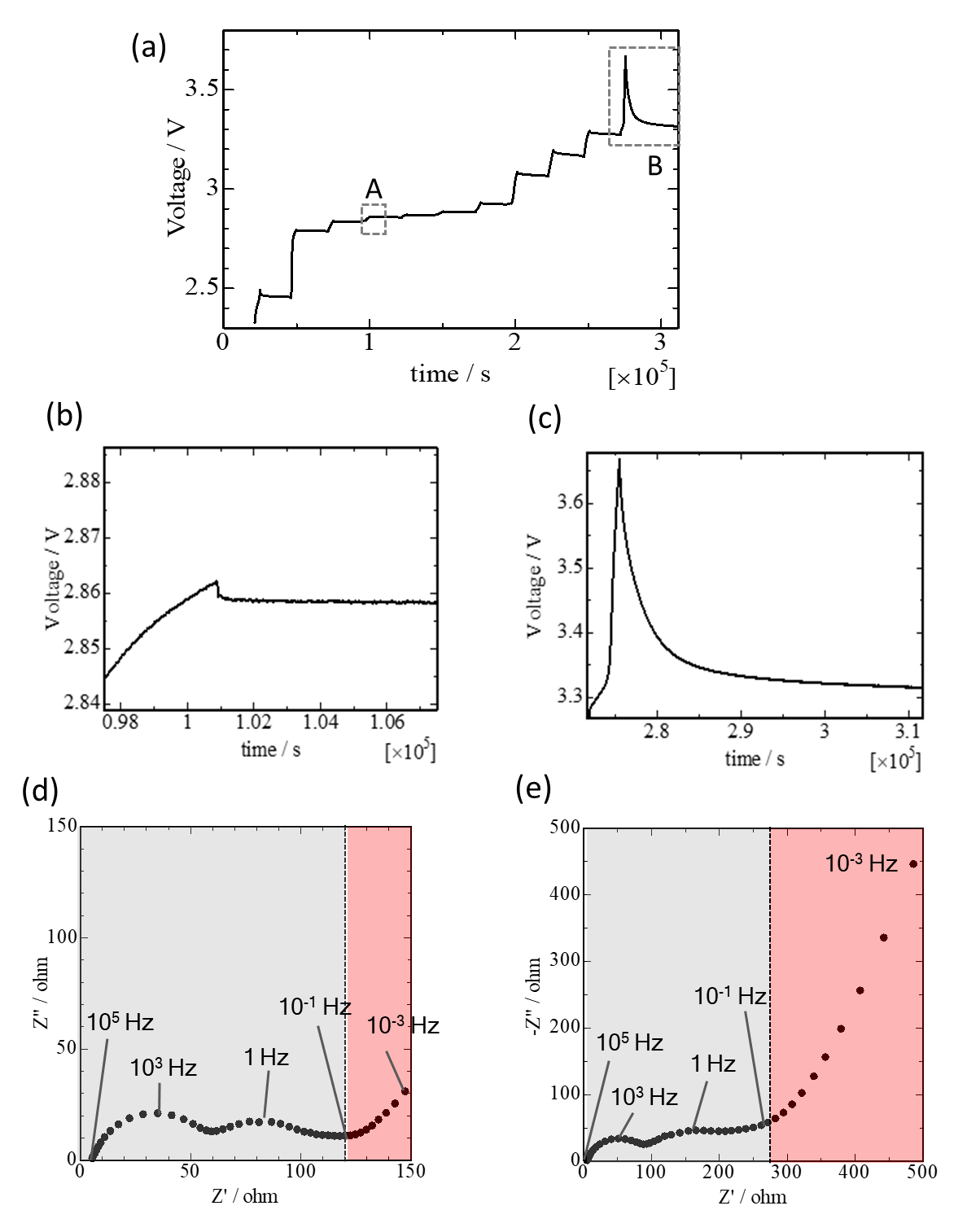


Supporting Figure S6 (a) Time-dependent voltage change in GITT curves during the charging process of cells using the Na_2_V_3_O_7_ electrode. The magnified views in regions A and B in (a) are shown in panels (b) and (c), respectively. Panels (d) and (e) show Nyquist plots measured after the end of relaxation of regions A and B, respectively.
